# Editorial: Fungal green biotechnology and biomanufacturing

**DOI:** 10.3389/fmicb.2023.1334993

**Published:** 2023-11-29

**Authors:** Gen Zou, Yongjun Wei, Xiao-Jun Ji

**Affiliations:** ^1^Shanghai Key Laboratory of Agricultural Genetics and Breeding, Institute of Edible Fungi, Shanghai Academy of Agricultural Sciences, Shanghai, China; ^2^School of Pharmaceutical Sciences, Laboratory of Synthetic Biology, Food Laboratory of Zhongyuan, Zhengzhou University, Zhengzhou, China; ^3^State Key Laboratory of Materials-Oriented Chemical Engineering, College of Biotechnology and Pharmaceutical Engineering, Nanjing Tech University, Nanjing, China

**Keywords:** fungi, synthetic biology, biotechnology, biomanufacturing, sustainability

Fungi are extremely widespread organisms on Earth and play significant roles in the environment and medicine. Many fungi are free-living in soil or water, while the others established parasitic or symbiotic relationship with plants or animals. Fungi have evolved the capability to synthesize diverse enzymes and natural products (Wei et al., 2019, 2021; Guan et al., 2020). Some of these products are commercially produced, while the others have potential applications in biotechnology. These highlight the tremendous potential of fungi for sustainable and green biomanufacturing (Zou et al., 2023). Recently, the potential of fungi-based biotechnology and biomanufacturing has been explored as a strategy to reduce the carbon emissions and achieve carbon neutrality (Gong et al., 2023). In order to further contribute to the emerging fields of fungal biotechnology, we have organized the Research Topic of “*Fungal green biotechnology and biomanufacturing*”.

This Research Topic focuses on the diverse and complex world of fungal biomanufacturing, exploring strategies for optimizing biotechnology approaches for yeasts, molds, and mushrooms. This includes using fungal cell factories to produce enzymes, natural products, bulk chemicals, biocontrol agents (BCAs), and fungal-based alternative food products. In addition, fungi-based biomanufacturing involves optimizing strain and fermentation processes. The articles collected in this Research Topic aim to shed light on the crucial role of fungal biotechnology and biomanufacturing play in advancing sustainable development.

The most frequently mentioned aspect of the fungal biotechnology is undoubtedly the use as BCAs. Biocontrol methods harness the power of natural enemies and antagonistic microorganisms to combat pests and pathogenic fungi (Li et al., 2021). These methods offer the advantage of being safe with low risks of pest resistance, making them popular in agricultural production. *Trichoderma harzianum*, a widely recognized BCAs, exhibits efficacy against a wide range of plant pathogens, such as *Fusarium*. Xiao et al. summarized studies on enhancing the biocontrol ability of *T. harzianum* through strain improvement. Strategies such as increasing the production of antimicrobial biomolecules and enhancing environmental adaptability have proven highly effective in strengthening its biological control capabilities. Another intriguing aspect of fungal biotechnology is the utilization of endophytic fungi, which colonizes the inner tissues of plants without causing disease symptoms. This mutualistic interaction provides benefits to both the host plants and the fungi. Many plant endophytic fungi show significant potential in biocontrol against plant diseases. Feng et al. report on the biocontrol abilities of *Chaetomium globosum*, a common plant endophytic fungus, against Fusarium crown rot (FCR) caused by *Fusarium pseudograminearum*. *C. globosum* modulates the rhizosphere microbiome, often referred to as the plant's second genome, and enhances the plants' resistance to FCR.

Fungi are capable of synthesizing a variety of valuable bulk chemicals, including organic acids and bioethanol. Recent advancements in genetic and metabolic engineering techniques, as well as the availability of sequenced genomes have greatly helped to harness their potential for the production of bulk chemicals (Zou et al., 2021). However, genetic modification of chassis cells, e.g., introducing pathways for using xylose and lignocellulosic hydrolysates to *Saccharomyces cerevisiae*, plays an essential role in facilitating cellulosic ethanol production. Wang et al. evaluated the fermentation performance of a modified *S. cerevisiae* strain GRE3^OE^ (with GRE3 overexpression) using pretreated substrates, including sweet sorghum bagasse, sorghum straw, and xylose hydrolysate as substrates. The study proved that GRE3^OE^ has the potential to be used as a candidate strain for industrial ethanol production using various lignocellulosic hydrolysates.

A common strategy in a synthetic biology effort for natural product biosynthesis is to build heterologous biosynthetic gene clusters in chassis cells (Xiao et al., 2023). However, this strategy is limited to situations where the biosynthetic pathway is elucidated and characterized. Microbial transformation refers to the process of using microorganisms or their enzymes to convert substrates into structurally related products. This approach has long been employed to produce natural product derivatives and is not relied on the knowledge of defined biosynthetic pathways. Peng et al. use *Beauveria bassiana*, one of the best-known species of entomopathogenic fungi, to produce stilbene methylglucoside with GPR119 agonistic activity. The results demonstrated that microbial biotransformation has the advantages, including highly regioselectivity and stereoselectivity, environmental sustainability, mild reaction conditions, and a straightforward production process.

The rapid growth of human civilizations has led to the development of new food products with increased nutritional characteristics and decreased environmental footprints. Fungi, a group of microorganisms that have been utilized in various foods for thousands of years, have recently gained significant attention in both research communities and commercial ventures. This interest is derived from the exploration of innovative applications in a diverse array of food products like alternative proteins. Technological advances in the cultivation and processing of fungi have created new possibilities for the control of textures, flavors, and nutritional properties of fungi-based foods (Ji et al., 2014). Qin et al. highlighted the technological advances in biotechnological production of omega-3 fatty acids. Biosynthesis of omega-3 fatty acids via engineered fungal cell factories remains the best solution to achieve a stable, sustainable, and affordable production strategy (Ji et al., 2015).

This Research Topic aims to summarize the advances in fungal biotechnology and biomanufacturing. However, several significant aspects have not been addressed: (1) Application of filamentous fungi in the production of industrial enzymes; (2) Research on the role of fungi in environmental pollution control; (3) The development of new building materials and vegan leathers using fungal mycelium; (4) In particular, mushrooms, the important agricultural fungi. Further exploration and discovery of fungal roles and applications in green biotechnology and biomanufacturing are expected to continue to unveil new insights in these areas.

## Author contributions

GZ: Writing – original draft. YW: Writing – review & editing. X-JJ: Writing – review & editing.
